# Development and psychometric testing of a new instrument to measure factors influencing women’s breast cancer prevention behaviors (ASSISTS)

**DOI:** 10.1186/s12905-016-0318-2

**Published:** 2016-07-22

**Authors:** Maryam Khazaee-Pool, Fereshteh Majlessi, Ali Montazaeri, Tahereh Pashaei, Ali Gholami, Koen Ponnet

**Affiliations:** Department of Health Education and Promotion, School of Health, Zanjan University of Medical Sciences, Zanjan, Iran; Department of Health Education and Promotion, School of Health, Tehran University of Medical Sciences, P.O. Box 15875-6951, Tehran, Iran; Mental Health Research Group, Health Metrics Research Center, Institute for Health Sciences Research, ACECR, Tehran, Iran; Department of Public Health, School of Health, Kurdistan University of Medical Sciences, Sanandaj, Iran; Department of Public Health, Neyshabur University of Medical Sciences, Neyshabur, Iran; Department of Epidemiology, School of Public Health, Iran University of Medical Sciences, Tehran, Iran; Department of Communication Studies and Sociology, University of Antwerp, Antwerp, Belgium; Higher Institute for Family Sciences, Odisee, Brussels, Belgium; Antwerp Maritime Academy, Antwerp, Belgium

**Keywords:** Breast cancer, Preventive behaviors, Instrument, Psychometrics, ASSISTS

## Abstract

**Background:**

Breast cancer preventive behaviors have an extreme effect on women’s health. Despite the benefits of preventive behaviors regarding breast cancer, they have not been implemented as routine care for healthy women. To assess this health issue, a reliable and valid scale is needed. The aim of the present study is to develop and examine the psychometric properties of a new scale, called the ASSISTS, in order to identify factors that affect women’s breast cancer prevention behaviors.

**Methods:**

A multi-phase instrument development method was performed to develop the questionnaire from February 2012 to September 2014. The item pool was generated based on secondary analyses of previous qualitative data. Then, content and face validity were applied to provide a pre-final version of the scale. The scale validation was conducted with a sample of women recruited from health centers affiliated with Tehran University of Medical Sciences. The construct validity (both exploratory and confirmatory), convergent validity, discriminate validity, internal consistency reliability and test-retest analysis of the questionnaire were tested.

**Results:**

Fifty-eight items were initially extracted from the secondary analysis of previous qualitative data. After content validity, this was reduced to 49 items. The exploratory factor analysis revealed seven factors (Attitude, supportive systems, self-efficacy, information seeking, stress management, stimulant and self-care) containing 33 items that jointly accounted for 60.62 % of the observed variance. The confirmatory factor analysis showed a model with appropriate fitness for the data. The Cronbach’s alpha coefficient for the subscales ranged from 0.68 to 0.85, and the Intraclass Correlation Coefficient (ICC) ranged from 0.71 to 0.98; which is well above the acceptable thresholds.

**Conclusion:**

The findings showed that the designed questionnaire was a valid and reliable instrument for assessing factors affecting women’s breast cancer prevention behaviors that can be used both in practice and in future studies.

## Background

Cancer is now the leading cause of death worldwide. It has a social impact on patients’ lives [1]. In addition, breast cancer is an increasingly global public health problem that has noticeable influences on the daily activities of patients. It is the most common type of cancer among females and the leading cause of cancer death in women [2]. In Iran, breast cancer is the cancer that is most frequently diagnosed in women. The literature shows that it affects Iranian women about one decade earlier than women in developed countries [3]. The incidence rate of breast cancer in Iranian women is 24.6 % of all cancers, and most of the women (67.6 %) are between 35 and 60 years of age [4]. Several risk factors may increase the chance of developing breast cancer, and lifestyle factors have a major effect on this field. Therefore, it can be reasoned that an effective approach to decrease the burden of breast cancer is prevention. It has been proposed that a suitable procedure for breast cancer prevention is preventive behaviors such as healthy lifestyle and screening [5], because there is evidence that increased physical activity due to positive lifestyle changes might help to prevent breast cancer and reduce the incidence of breast cancer [6]. Lifestyle changes include increased intake of healthy diet, decreased alcohol consumption and increased exercise [7–9].

Screening behaviors such as breast self-examination (BSE), mammography and clinical breast examination (CBE) are also considered to be a valuable method of early detection and a way to reduce mortality rates [10]. However, by our own experiences, we observed that most Iranian women do not perform breast cancer screening behaviors because the Iranian Ministry of Health does not offer any national population-based screening programs for women. Few studies have considered behaviors related to breast cancer prevention in Iranian women [11, 12]. To make changes happen, understanding individuals’ health behaviors in regard to specific health issues is essential. Reviews on health-related behaviors have indicated that women will commonly not attempt to take preventive measures unless they have slight levels of related support, motivation and information [13, 14]. In addition, studies have shown that persons will be more likely to take part in the suggested behaviors if they improve their self-efficacy abilities to change their unhealthy behaviors [15, 16].

As a result, in order to develop effective interventions for improving breast cancer preventive behaviors, the predictive factors of these behaviors need to be recognized. At present, there exists no comprehensive, validated questionnaire on this topic. Thus, the purpose of the current paper was to develop and examine the psychometric properties of a newly developed instrument, called the ASSISTS, that can be used to explore factors influencing Iranian women’s behaviors for breast cancer prevention and perhaps show areas for applying interventions to increase preventive behaviors among women. To establish the validity of our instrument, the relationship between the scale scores of our instrument will be associated with the scores of four potentially associated constructs, namely perceived social support, cancer attitude, self-efficacy and stress management with regard to promoting a healthy lifestyle.

## Methods

### Research design

This study was approved by the Ethics Committee of Tehran University of Medical Sciences [Grant number 22847] and all participants completed informed written consent. The study was conducted in two phases. In the first phase, we started by generating items and developing the instruments. A secondary analysis of previous qualitative data [11] was done to provide an initial indication of candidate items, to generate relevant items, to evaluate face and content validity, and to determine the most appropriate phrasing.

The second phase was a testing phase, involving cross-sectional studies with women. We carried out both exploratory factor analysis and confirmatory factor analysis, and tested the convergent and discriminant validity and the internal consistency of the scale. Thereafter, test-retest reliability was examined using an independent sample of 25 women.

### Phase 1: item generation and scale development phase

This study was carried out to develop a scale for measuring factors influencing women’s breast cancer prevention behaviors. Items were derived from secondary analysis from a previous qualitative research conducted by Khazaee-Pool in which Iranian women’s experiences about breast cancer preventive behaviors were explored [11]. Based on the secondary analysis, by Graneheim method [17], five main themes and 29 subthemes were considered to be key factors relating to breast cancer preventive behaviors. The framework is provided in Table 1. The item pool contained 97 items at this point. The content of the items was made clear, and extra items were omitted through discussion. The main investigator and other researchers read items and removed extra items. Finally, the first draft of the scale was developed and consisted of 58 items. Each item was rated on a five-point response scale anchored at 1 = *never* to 5 = *always*. Thereafter, content and face validity were examined to develop the pre-final version of the instrument.

**Table 1:** Themes and sub-themes identified by secondary analysis of previous data (phase 1)

Themes	Sub-themes
Attitudes toward breast cancer and prevention	
	Superstitious beliefs
	Fatalism
	Prejudice
	Worries
	Feelings of giving up
	Sense of shame
Capability in breast cancer prevention	
	Motivation for changing behavior
	Previous positive experiences
	Self-responsibility
	Self-esteem
	Competence and worthiness
Self-care	
	Healthy lifestyle
	Self-monitoring
	Positive thinking
	Relaxation
	Spirituality
Social support	
	Family and friends support
	Health care system support
	Support from government and policy makers
	Insufficient family support
	Lack of resources and facilities
	Weaknesses of strategies and policies
Information seeking	
	Media
	Public education
	Intersectional cooperation
	Interpersonal interactions
	Inattention to individual needs
	Stereotypical training
	Insufficient information

#### Content validity

Both qualitative and quantitative content validity were examined. In the qualitative stage, a scientific expert panel (i.e., a team of investigators specialized in health education, breast cancer and psychometrics) assessed the content validity of the scale. The expert panel evaluated the wording, grammar, item allocation and scaling of the scale. In the quantitative stage, both the content validity index (CVI) and the content validity ratio (CVR) were calculated. The clarity, simplicity and relevance of each item were measured by the CVI [18, 19]. In order to calculate the CVI, a Likert-type ordinal scale with four possible responses was applied. The answers were rated from 1 = *not relevant, not simple and not clear* to 4 = *very relevant, very simple and very clear*. The CVI was assessed as the proportion of items that received a rating of 3 or 4 by the experts [20]. A CVI score below .80 for an item was not acceptable [21]. The CVR tested the essentiality of the items. To assess the CVR, the expert panel scored each item as 1 = *essential*, 2 = *useful but not essential*, or 3 = *not essential* [20]. Then, based on the Lawshe Table [22], items with a CVR score of 0.62 or above were considered to be acceptable and were retained.

In the quantitative stage, items with a CVR and a CVI less than .62 and .80, respectively, were deleted. In total, 9 items were deleted, resulting in a 49-item pool. The expert panel also revised the instrument with regard to grammar, wording and item allocation. For example, the sentence “Breast cancer destroys my femininity” was changed to “If I get breast cancer, my feminine identity will be lost”. The 49-item pool remained in the analyses below and consisted of positively worded and negatively worded statements with five response options: 1 = *never*, 2 = *rarely*, 3 = *sometimes*, 4 = *often*, and 5 = *always.*

#### Face validity

Both qualitative and quantitative methods were used to assess face validity. A group of women (*n* = 10) were asked to evaluate each item of the questionnaire and to indicate if they felt ambiguity or difficulty in replying to the Iranian version of the ASSISTS questionnaire. Based on the participants’ viewpoints, the ambiguous items were adapted. In a quantitative phase, the impact score (frequency × importance) was assessed to show the percentage of women who identified each item as important or quite important on a five-point Likert scale. Items were considered to be appropriate if they had an impact score equal to or more than 1.5 (which corresponds to a mean frequency of 50 % and a mean importance of three on the five-point Likert scale) [23]. In conclusion, all items had an impact score higher than 1.5. The range of impact score was from 1.9 to 5. None of the items were omitted, and the first form of the questionnaire containing 49 items was established for the next phase of psychometric evaluation. In other words, the group of women indicated that they experienced no difficulties reading and understanding the 49 items.

### Phase 2: testing phase

#### The main study and the data collection

In order to test the psychometric properties of the ASSISTS scale in a wider setting, a cross-sectional study was designed to be carried out in Tehran, Iran, from February 2012 to September 2014. A multistage cluster sampling was used. Firstly, Tehran (the capital of Iran) was separated into five areas: north, south, west, east and central. All health centers located in these five areas that were affiliated to the Tehran University of Medical Sciences were recognized. Then five health centers in each area were randomly chosen. Participants who visited health centers affiliated to Tehran University of Medical Sciences were entered into the study if they were 30 years old or older, literate and healthy (i.e., having no history of breast cancer) and wanted to take part in the study. After the first author conducted a short interview and provided information about the aim of the study, women who accepted to participate in the study completed the ASSISTS scale. Besides the study scale, the demographic characteristics of participants including employment status, educational level and marital status were also collected. In order to collect data, educated investigators performed face-to-face interviews.

#### Measures

To establish the validity of the ASSIST, we also administered the following scales from a group of women: The Multidimensional Scale of Perceived Social Support, the Cancer Attitude Scale, the Generalized Self-Efficacy Scale and the Stress Management Scale with regard to a health-promoting lifestyle.

The Multidimensional Scale of Perceived Social Support (MSPSS) is a brief instrument developed to assess perceptions of support from three sources: family, friends and a significant other. The MSPSS comprises a total of 12 items, with four items for each of three subscales. Each item was valued on a seven-point Likert-type scale, ranging from 1 = *very strongly disagree* to 7 = *very strongly agree* [24]. In several studies, the MSPSS has been presented to have good internal and test-retest reliability, good validity and a fairly stable factorial structure. It has been translated into many languages, including Farsi (Persian) [25]. The minimum and maximum scores of the questionnaire are 12 and 84, respectively. A higher score indicates greater perceived social support. A score of 65 or less is considered the cutoff point for eligibility of services. The Cronbach’s alpha coefficient for the total scale was .81, indicating good reliability in our sample.

The Cancer Attitude Scale (CAS) is an Iranian validated questionnaire with 15 items assessing attitudes toward cancer. It has two domains, senses and beliefs (9 items) and worries (6 items). The items were rated on a five-point Likert-type scale, anchored at the extremes with 1 = *completely agree* to 5 = *completely disagree*. All items were scored in the direction of a negative attitude, with higher scores indicating more negative attitudes toward cancer and preventive behaviors. A minimum score is 15, and 75 is the maximum [26]. The Cronbach’s alpha coefficient for the CAS was .84 in our sample.

The Generalized Self-Efficacy Scale (GSE-10) is a 10-item scale developed by Schwarzer [27]. This scale assesses self-efficacy based on subjects’ propensities that correlate to emotion, optimism and work satisfaction. It is a self-report measure of self-efficacy, rated on a four-point experience scale ranging from 1 = *not at all true* to 4 = *exactly true*. Total self-efficacy score is derived from all 10 items and ranges from 10 to 40, with higher scores indicating higher self-efficacy. This questionnaire has been confirmed to have good validity and reliability [27, 28]. The present study also found a Cronbach’s alpha of .76 for the total score.

The Health Promoting Lifestyle-II (HPLP II) assesses individuals’ health-promoting behaviors based on Pender’s health promotion model. It is a 52-item instrument that yields a multidimensional profile of scores across six domains: nutrition (9 items), physical activity (8 items), interpersonal relations (9 items), stress management (8 items), health responsibility (9 items) and spiritual growth (9 items). In this study we have only used the stress management subscale of the instrument. The total score for the HPLP-II stress management subscale ranges from 8 to 32. A higher score indicates more stress management. Each item was estimated on a four point Likert-type measure, with 1 = *never*, 2 = *sometimes*, 3 = *often*, and 4 = *always*. The Cronbach’s alpha coefficient for the HPLP-II subscale was .70 in our sample.

##### Statistical analysis

Several statistical methods were applied to test the psychometric properties of the scale. These are presented as follows.

### Validity

#### Construct validity

After the item analysis, the 49 remaining items were used to estimate the construct validity using exploratory factor analysis (EFA) and confirmatory factor analysis (CFA). Furthermore, both convergent validity and divergent validity were assessed.

##### Exploratory factor analysis

EFA was applied to specify the main factors of the questionnaire. We estimated the sample size a priori. As recommended by Gable and Wolf, a sample of five to ten women per item is necessary in order to ensure a conceptually clear factor structure for analysis [30]. The desired minimum required sample size was thus determined to be 250 women. These women were recruited from the health centers (see data collection section). A principal component analysis (PCA) with varimax rotation was used to extract the main factors. The Kaiser-Meyer-Olkin (KMO) measure and Bartlett’s test of sphericity were applied to assess the adequacy of the sample for the factor analysis [31]. Any factor with an eigenvalue above 1 was considered significant for factor extraction, and a scree plot was used to specify the number of factors. Factor loadings equal to or greater than .40 were considered acceptable [32].

##### Confirmative factor analysis

A confirmatory factor analysis was applied in order to assess the coherence between the data and the structure. Considering the possible attrition related to test-retest analysis, we planned to recruit a separate sample of 130 women from health centers affiliated to Tehran University of Medical Sciences. Assigning four individuals to each item, a sample size of 130 was estimated [33]. The model fit was evaluated using multiple fit indices. As suggested, various fit indices measuring relative Chi-square, Goodness of Fit Index (GFI), Comparative Fit Index (CFI), Root Mean Square Error of Approximation (RMSEA), Non-Normed Fit Index (NNFI), Normed Fit Index (NFI) and Standardized Root Mean Square Residual (SRMR) were taken into account [34, 35]. The GFI, CFI, NNFI and NF range between 0 and 1 [36], but values of 0.90 or above are commonly indicated as acceptable model fits [34]. An RMSEA value between .08 and .10 demonstrates an average fit, and a value below .08 shows a good fit. Values below .05 indicate a good fit for SRMR, but values between .05 and .08, and between .08 and .10 indicate a close fit or are acceptable, respectively [37].

##### Convergent & divergent validity

To assess convergent and divergent validity, a new sample of 180 women aged 30 or above was recruited. Table 2 provides the descriptive characteristics of the 180 women. Apart from the ASSISTS, the women also completed the Iranian validated versions of the MSPSS [24, 25], CAS [26], GSE [27, 28], and the stress management subscale of the HPLP-II [29].

**Table 2:** Characteristics of the study sample

		EFA sample (*n* = 250)	CFA sample (*n* = 130)	Convergent validity sample (*n* = 180)	Test-retest sample (*n* = 25)
		Number (%)	Number (%)	Number (%)	Number (%)
Age (years)					
	30–34	31 (12.4)	27 (20.77)	15 (8.33)	6 (24)
	35–39	70 (28)	49 (37.7)	10 (5.6)	4 (16)
	40 and above	149 (59.6)	54 (41.53)	155 (86.07)	15 (60)
	Mean (SD)	41.25 (6.34)	39.47 (5.62)	53 (8)	43.19 (8.61)
	Range	30–72	30–65	34–73	30–57
Employment status					
	Housewife	144 (57.6)	86 (66.15)	117 (65)	11 (44)
	Employed	106 (42.4)	44 (33.85)	63 (35)	14 (56)
Educational Level					
	Primary	24 (9.6)	18 (13.85)	32 (17)	2 (8)
	Secondary	114 (45.6)	80 (61.54)	79 (43)	9 (36)
	Higher	112 (44.8)	32 (24.61)	69 (38)	14 (56)
Marital status					
	Single/divorced/widowed	60 (24)	34 (26.15)	51 (29)	9 (36)
	Married	190 (76)	96 (73.85)	129 (71)	16 (64)

We first assessed the item-convergent validity by examining the correlations between the item scores and the subscale scores of the ASSISTS by use of the Spearman correlation coefficient. We expected that, for each subscale of the ASSISTS, the item scores of the subscale (e.g., self-care) would correlate more with the total score of the respective subscale (e.g., self-care), rather than the total score of other subscales (e.g., stress management). Correlation values between 0 and .20 are considered poor; between .21 and .40, fair; between .41 and .60, good; between 0.61 and 0.80, very good; and above .81, excellent. [38]. Item-convergent validity exists when an item has a significantly higher correlation with its own scale compared with the other scales, and item divergent validity exists when an item has lower correlation with other scales [39]. Then we evaluated convergent and divergent validity of four subscales of the ASSISTS (stress management, attitudes, supportive system and self-efficacy) compared to the abovementioned validated questionnaires. For three subscales of the ASSISTS (self-care, motivation and information seeking) we were unable to assess convergent validity due to the lack of suitable dimensions or Iranian validated scales. Convergent validity is established when a subscale of the ASSISTS correlates moderately with the validated questionnaire (correlation .21 or above). We expected moderate correlations between the stress management subscale of the ASSIST and the stress management subscale of the HPLP-II, between the attitude subscale of the ASSIST and the CAS, between the supportive system subscale of the ASSIST and the MSPSS, and between the self-efficacy subscale of the ASSISTS and the GSE-10. A poor correlation (.20 or lower) between a subscale of the ASSISTS and one of the validated questionnaires demonstrates divergent validity.

### Reliability

#### Internal consistency

Cronbach’s alpha coefficient was applied to assess the internal consistency of each item, the whole questionnaire and each dimensions of the ASSISTS questionnaire. The alpha values equal to .70 or higher were considered acceptable [33, 40].

#### Test-retest

The test-retest reliability was applied to examine the questionnaire’s stability by estimating the intraclass correlation coefficient (ICC). The scale was re-administered to 25 women two weeks after the first completion. ICC values of .40 or above are considered acceptable [41]. All statistical analyses, except confirmatory factor analysis, were performed using SPSS 18.0 [42]. The confirmatory factory analysis was performed using LISREL 8.80 [43].

## Results

### Construct validity

#### Exploratory factor analysis

**Fig. 1:**
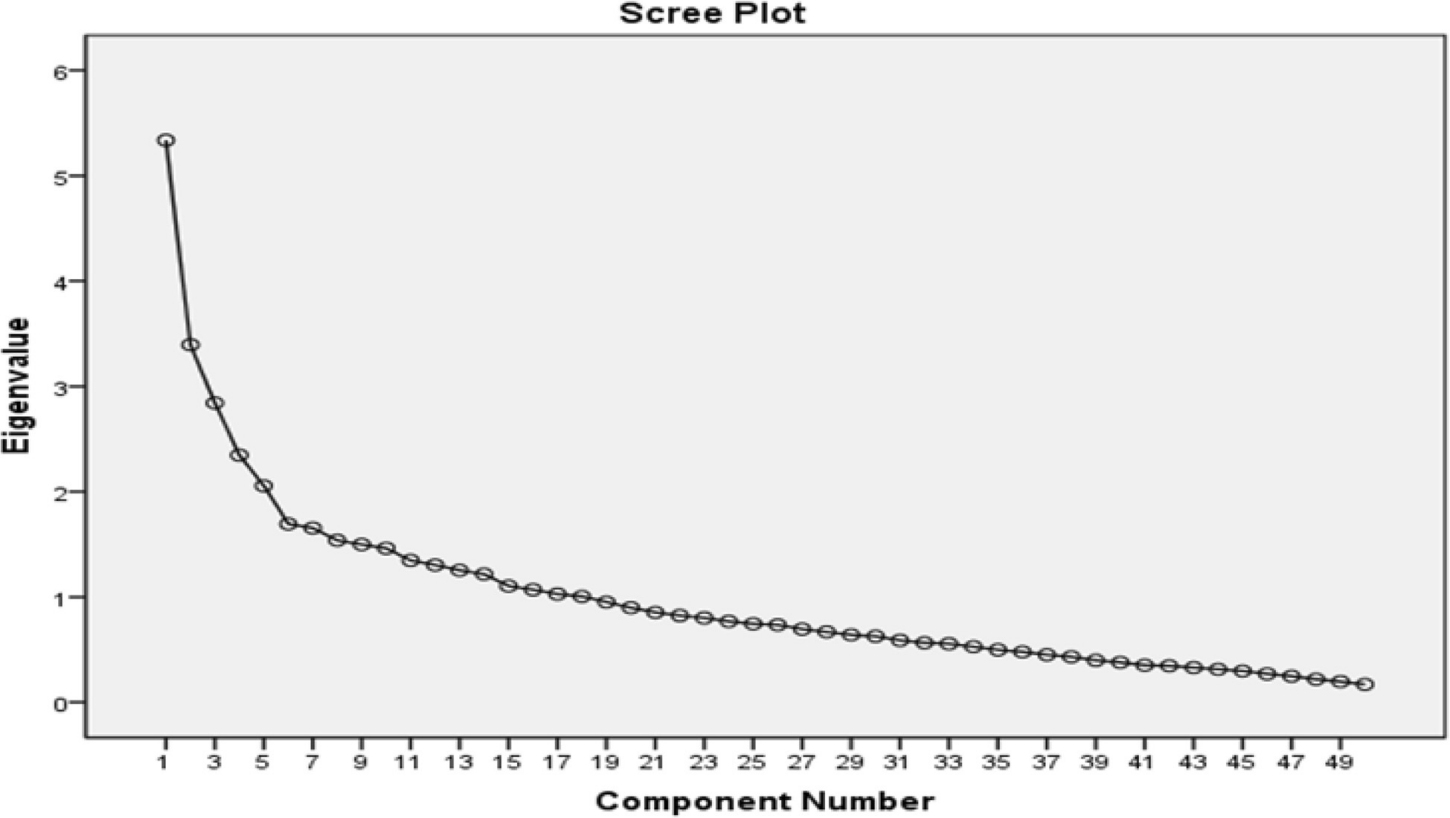
Scree plot for determining factors of the designed instrument

The Kaiser-Meyer-Olkin measure was .733, and the Bartlett’s test of sphericity was significant (*χ*2 = 2180.98, *p* < .001), indicating adequacy of the sample for EFA. Initially, for the 49-item scale, 13 factors showed eigenvalues above 1.0, explaining the 66.34 % variance. However, the scree plot showed a 7-factor solution (Fig. 1). This factor solution was explored by repeatedly assessing the item performance with elimination of the items in a step-by-step process. After eliminating the items with factor loadings below .40, we obtained a final factor solution that consisted of a 33-item questionnaire loading on seven distinct constructs. These constructs jointly accounted for 60.62 % of the observed variance.

As shown in Table 3, seven factors were found: Factor 1 (supportive systems) included 5 items (items 10, 11, 12, 13 and 14), factor 2 (self-efficacy) included 3 items (item 7, 8 and 9), factor 3 (self-care) included 7 items (items 24, 25, 26, 27, 28, 29 and 30), factor 4 (stress management) included 3 items (items 31, 32 and 33), factor 5 (motivation) included 3 items (items 4, 5 and 6), factor 6 (information seeking) included 4 items (items 15, 16, 17 and 20) and factor 7 included 8 items (items 1, 2, 3, 18, 19, 21, 22 and 23). We refer to Appendix for the items of the ASSISTS.

**Table 3:** Exploratory factory analysis of the ASSISTS (*n* = 250)

Item	Factor 1	Factor 2	Factor 3	Factor 4	Factor 5	Factor 6	Factor 7
10	**0.840**	0.322	−.002	0.241	0.321	0.260	0.351
11	**0.886**	0.046	0.071	0.307	0.218	0.173	0.001
12	**0.872**	−0.082	−0.044	0.202	0.319	0**.**293	0.185
13	**0.852**	0.086	0.252	0.125	0.213	0.360	0.230
14	**0.800**	0.052	−.021	0.239	0.034	0.293	0.102
7	0.053	**0.882**	0.006	0.375	0.241	0.123	0.074
8	−0.160	**0.812**	−0.310	0.252	0.182	0.311	−0.215
9	0.24	**0.787**	0.191	−0.311	0.271	0.318	0.274
24	−0.137	0.43	**0.849**	0.226	0.258	0.231	−0.187
25	0.057	0.284	**0.831**	0.143	0.083	0.128	0.218
26	0.319	0.082	**0.887**	−0.466	0.290	0.229	0.339
27	0.250	0.161	**0.722**	−0.370	0.312	0.345	0.212
28	0.003	−0.071	**0.855**	−0.268	0.022	0.288	0.255
29	0.050	0.160	**0.879**	−0.171	0.156	0.203	−0.196
30	0.125	0.121	**0.708**	0.183	0.128	0.398	0.351
31	0.129	−0.368	0.132	**0.845**	0.059	−0.259	0.140
32	−0.412	−0.266	0.148	**0.798**	0.305	0.348	−0.131
33	0.218	−0.270	0.240	**0.785**	0.352	0.189	0.245
4	0.165	0.382	−0.005	0.195	**0.784**	0.289	0.394
5	−0.129	0.239	−0.079	−0.089	**0.789**	0.376	0.429
6	−0.112	−0.347	0.082	−0.039	**0.791**	0.243	0.183
15	0.350	−.028	0.002	0.198	0.164	**0.870**	−0.029
16	0.286	0.094	0.271	0.374	0.211	**0.827**	−0.069
17	0.050	0.014	0.315	0.206	0.309	**0.754**	0.249
20	0.426	0.115	0.274	0.043	0.222	**0.704**	0.267
1	−0.026	−0.070	0.084	−0.141	0.170	0.164	**0.754**
2	0.127	0.282	−0.053	0.151	0.322	0.343	**0.662**
3	−0.085	0.136	0.031	0.076	0.177	0.291	**0.721**
18	0.129	−0.003	0.254	0.068	0.188	0.355	**0.652**
19	−0.312	−0.154	0.188	0.052	0.378	0.137	**0.667**
21	0.125	0.317	0.267	−0.171	0.218	0.288	**0.763**
22	0.229	0.256	0.211	0.022	0.203	0.321	**0.646**
23	0.173	0.238	0.192	0.140	0.311	0.188	**0.644**

*Note:* Figures in bold are related to factor loadings equal to or greater than 0.40

#### Confirmatory factor analysis

**Fig. 2:**
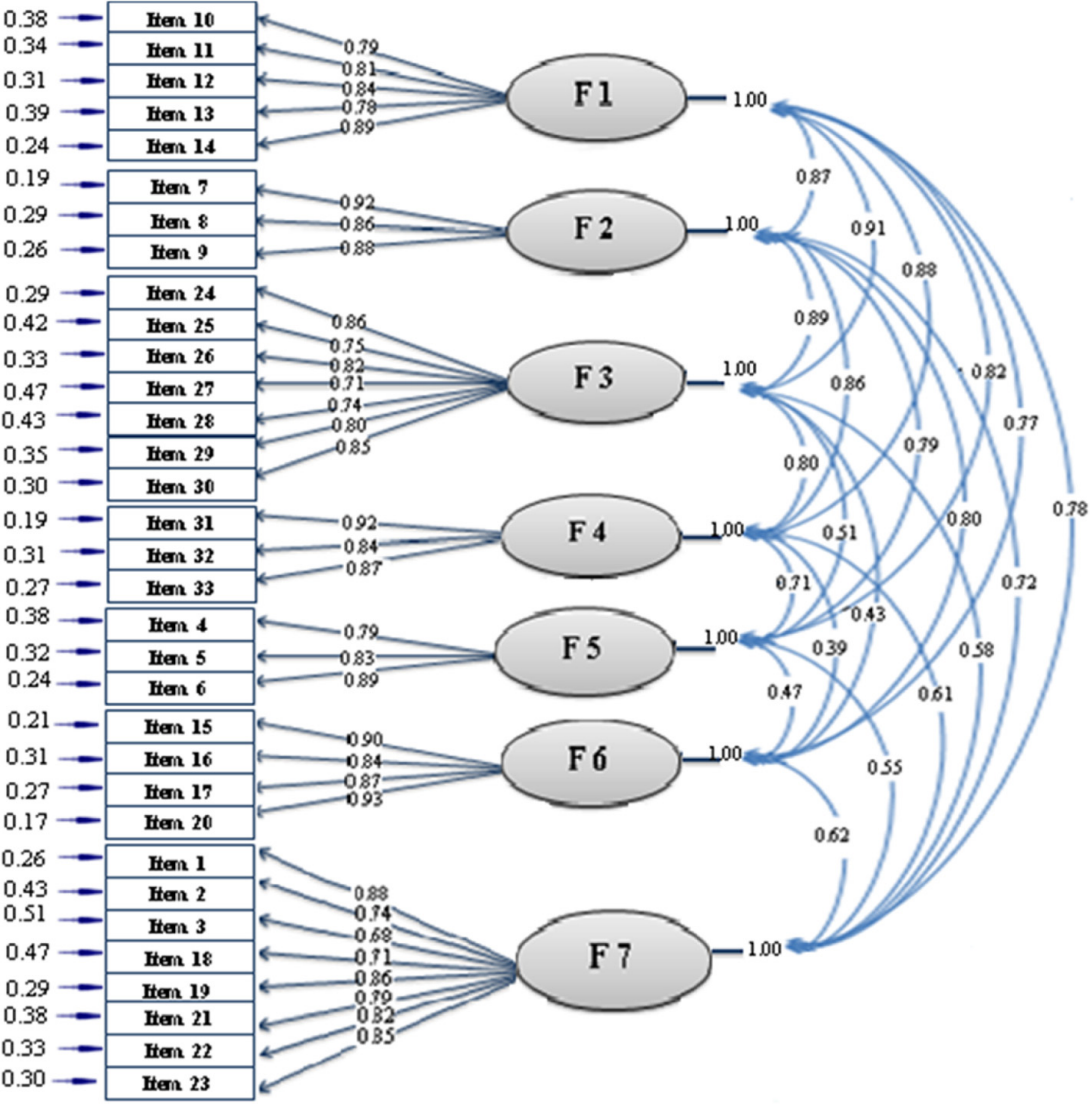
A seven-factor model for the questionnaire obtained from confirmatory factory analysis (*n* = 130)

We conducted a confirmatory factor analysis on the 33-item questionnaire to test the fitness of the model obtained from the EFA. Figure 2 shows the best model fit. Covariance matrixes were used and fit indexes were calculated. All fit indices proved to be good. The relative chi-square (*χ*2/df) was equal to 1.86 (*p* < .001). The RMSEA of the model was .031 (90 % CI = .021 – .089), and the SRMR was .030. All comparative indices of the model, including GFI, AGFI, CFI, NNFI and NFI, were more than .90 (.99, .98, .94, 1.00 and .98 respectively).

#### Convergent-divergent and concurrent validity

Table 4 presents the item-convergent validity for the ASSISTS scale. As can be seen, all coefficients are higher than .20, and most of them are higher than 0.40. Self-care and self-efficacy had the lowest and the highest item-convergent validity, respectively (Table 4). Convergent validity was assessed by the correlation between the different subscales of the ASSISTS and the MSPSS, the CAS, the GSE and the stress management subscale of the HPLP-II. The correlation between the stress management subscale of the ASSISTS and the HPLP-II was .65, which indicated that the convergent validity was very good. Likewise, the correlations between the attitudes, supportive systems and self-efficacy of the ASSISTS and the CAS, MSPSS and GSE, respectively, were between .42 and .45, indicating a good convergent validity. The other correlations were low (≤ .20), indicating that the divergent validity was good (Table 5).

**Table 4:** Item-scale correlation matrix for the seven ASSISTS measures (*n* = 180)

ASSISTS dimensions							
	SS	SE	SC	SM	ST	IS	AT
SS (item number)							
Item 10	**0.56**	0.45	0.41	0.04	0.31	0.23	0.13
Item 11	**0.74**	0.25	0.22	−0.02	0.18	0.21	0.15
Item 12	**0.57**	−0.16	−0.09	−0.03	−0.11	−0.04	0.03
Item 13	**0.50**	0.07	−.008	0.15	−0.02	0.13	0.05
Item 14	**0.48**	0.19	0.10	−0.007	0.11	0.30	0.17
SE (item number)							
Item 7	0.14	**0.67**	0.20	0.13	0.10	0.24	0.13
Item 8	0.004	**0.60**	0.17	0.07	0.13	0.13	0.21
Item 9	0.17	**0.61**	0.18	0.05	0.25	0.21	0.24
SC (item number)							
Item 24	0.03	0.1	**0.31**	−0.04	0.02	0.07	0.008
Item 25	0.03	0.06	**0.36**	0.16	−0.06	−0.02	−0.15
Item 26	−0.03	−0.04	**0.21**	0.22	0.01	−0.05	−0.07
Item 27	0.06	0.13	**0.29**	0.07	0.02	0.12	−0.06
Item 28	0.12	0.06	**0.33**	−0.02	−0.08	0.00	0.01
Item 29	0.05	0.01	**0.35**	−0.19	0.06	0.09	0.28
Item 30	0.03	0.32	**0.28**	−0.05	0.18	0.09	0.23
SM (item number)							
Item 31	0.12	0.07	0.08	**0.42**	0.04	−0.11	0.04
Item 32	−0.07	0.04	0.06	**0.47**	0.03	0.15	0.06
Item 33	−0.01	0.06	−0.06	**0.45**	0.04	0.07	−0.03
MO (item number)							
Item 4	0.05	0.001	0.02	−0.08	**0.29**	0.08	0.07
Item 5	−0.01	0.18	0.06	0.02	**0.37**	0.14	0.02
Item 6	0.09	0.11	−0.02	0.15	**0.49**	0.01	0.16
IS (item number)							
Item 15	−0.04	0.15	0.07	0.05	−0.03	**0.41**	0.17
Item 16	0.06	0.04	−0.05	0.08	0.18	**0.37**	0.04
Item 17	0.23	0.15	0.05	−0.08	0.21	**0.43**	0.21
Item 20	0.13	0.13	0.13	0.05	−0.02	**0.31**	0.02
AT (item number)							
Item 1	−0.05	0.08	0.13	0.13	0.09	0.04	**0.51**
Item 2	0.09	0.28	0.06	0.16	0.18	0.21	**0.61**
Item 3	0.12	0.26	0.08	0.03	0.17	0.13	**0.53**
Item 18	0.1	0.11	−0.03	−0.05	0.07	0.16	**0.30**
Item 19	0.1	0.18	0.14	0.00	0.06	0.06	**0.33**
Item 21	0.09	0.07	−.03	0.20	0.09	0.21	**0.42**
Item 22	0.03	−0.05	0.06	−0.07	−0.07	0.06	**0.43**
Item 23	0.16	0.14	−0.02	−0.10	0.14	0.14	**0.41**

*Note: SS* supportive systems, *SE* self-efficacy, *SC* self-care, *SM* stress management, *MO* motivation, *IS* information seeking, *AT* attitudes

*Note:* The bold data reflect the higher item-scale correlation for the seven structures of ASSISTS questionnaire

**Table 5:** Correlations between some ASSISTS domains and other validated questionnaires

ASSISTS dimensions	Correlation with other validated questionnaires			
	HPLP-II (SS)	CAS	MSPSS	GSE
SM	**0.65**	0.04	0.08	0.04
AT	0.16	**0.42**	0.23	0.05
SS	0.03	0.10	**0.45**	0.13
SE	0.25	0.14	0.11	**0.44**
MO	0.20	0.18	0.27	0.23
IS	0.12	0.01	0.18	0.13
SC	0.06	0.01	0.15	0.06

*Note: SS* supportive systems, *SE* self-efficacy, *SC* self-care, *SM* stress management, *MO* motivation, *IS* information seeking, *AT* attitudes, *MSPSS* perceived social support, *CAS* cancer attitude scale, *GSE* general self-efficacy scale, *HPLP-II (SS)* Health Promoting Lifestyle-II (Stress Management Domain)

*Note:* The bold data reflected higher correlations between each ASSISTS domains and other validated questionnaires (HPLP-II, CAS, MSPSS, and GSE)

### Reliability

To measure the reliability, the Cronbach’s alpha was calculated separately for the ASSISTS as well as for each factor of the ASSISTS. The Cronbach’s alpha coefficient for the ASSISTS was .80 and ranged from .79 to .85 for its subscales, which is well above the acceptable threshold, with the attitude subscale as an exception, with alpha = .69. Thus, no items of the instrument were omitted in this phase. In addition, test-retest analysis was conducted to test the stability of the instrument. The results indicated satisfactory results. Intraclass correlation (ICC) was .86 for the ASSISTS and ranged from .80 to .93 (good to excellent) for the subscales of the ASSISTS, lending support for the stability of the instrument, with the exception of the Attitude subscale, which had an ICC value slightly below the threshold (.79). The results are presented in Table 6.

**Table 6:** Measures of internal consistency and stability

Factor	The name of factor	Number of items	Cronbach alpha (*n* = 250)	ICC (*n* = 25)
1	Supportive systems	5 items (10–14)	0.850	0.932
2	Self-efficacy	3 items (7–9)	0.827	0.898
3	Self-care	7 items (24–30)	0.819	0.874
4	Stress management	3 items (31–33)	0.809	0.859
5	Motivation	3 items (4–6)	0.788	0.857
6	Information seeking	4 items (15–17, 20)	0.787	0.803
7	Attitudes	8 items (1–3, 18, 19, 21–23)	0.689	0.789
Total		33 items	0.797	0.860

## Discussion

In this study, we described the development and psychometric properties of a new instrument, called the ASSISTS, for assessing factors that affect women’s breast cancer prevention behaviors. This is the first study to provide a measure for evaluating the factors associated with breast cancer preventive behaviors in Iranian women. The content of the instrument items was initially developed based on a secondary analysis of previous qualitative data [11] to ensure that this new instrument covered all theoretical concepts for breast cancer preventive behaviors. After exploratory factor analysis, a 7-domain instrument emerged. A confirmatory factor analysis revealed that the fit of the data was satisfactory. As such, the final 33-item ASSISTS instrument contained seven subscales (attitudes, support systems, self-efficacy, information seeking, stress management, self-care and motivation).

Items included in the attitudes and stimulant subscales reflect conditions that might encourage women to experience breast cancer preventive behaviors. The attitudes subscale can help practitioners because it includes factors that impede or facilitate preventive behaviors, including issues related to a woman’s personal concerns. It is recognized that some factors, like knowledge, beliefs, attitudes, values and personal priorities, can motivate people to perform and modify their behavior [44, 45]. The self-care, stress management, information seeking and self-efficacy subscales include issues referring to personal skills, abilities, behaviors and habits that induce women to engage or not to engage in preventive behaviors. The information seeking behavior subscale reflects the way people search for and apply both active and passive information. More specifically, it refers to women’s practices for gaining health information via various sources, such as family, media, healthcare personnel and other means. When women are aware of the importance of preventive behaviors, they will have greater motivation to perform such behaviors. Modifying behaviors, especially lifestyle behaviors, requires long-term investments. Thus, it is unlikely for women to accept such behaviors out of habit without any conscious decision to do so. In addition, the stress management subscale covers a wide range of approaches aimed at controlling women’s levels of stress, commonly for the purpose of enhancing everyday activities. For instance, a number of self-help approaches to stress prevention have been developed in the health centers affiliated to our university, such as relaxation, Quran reading, praying, positive thinking and establishing sleep and rest time.

Self-efficacy has a positive impact on health promoting behaviors and is associated with increasing breast cancer preventive behaviors, so self-efficacy is of great importance in the issue of behavioral change. It is important to know that women who had more positive expectations about breast cancer prevention felt more efficacious about practicing preventive behaviors in the face of barriers such as superstitious beliefs, prejudices, worries, feelings of giving up, sense of shame, lack of a health care facility, or things going wrong there. In other words, if one thinks he/she will get more benefit from behaving actively, this may be associated with better feelings of efficacy in the face of barriers, therefore increasing the chance of receiving the preventive behaviors. This is why it is discussed that preventive interventions must change women’s attitudes toward health and increase self-efficacy.

Items of the supportive systems subscale refer to factors that may facilitate maintenance, repetition and fixing of preventive behaviors. Support may come from family members, peers, healthcare workers, decision-makers and insurance systems. It is well-known that reinforcing behavior from other persons facilitates continuation, repetition and stabilization of behavior [44, 45]. However, the focus of the present study was to develop a scale containing the most important factors related to breast cancer preventive behaviors, namely lifestyle behaviors and self-care. It can be argued that by addressing these activities in women, it is also important to address their unmet needs for social support [46, 47]. In the present study, we believe that women need instrumental, informational and emotional support to perform preventive behaviors, and thus we included all aspects of social support. For instance, women who receive support from different sources (e.g., family, friends) are more likely to participate in breast cancer prevention behaviors. However, taking into account the different aspects of social support, one direction for future studies might be to examine more thoroughly which aspects of support have to be included.

Generally, the findings showed satisfactory psychometric properties for the scale. The CVI and the CVR showed that the content validity was reasonable. In addition, the results of the exploratory and confirmatory factor analyses showed a good structure for our new questionnaire. Exploratory factor analysis revealed that the seven-factor structure of the instrument accounted for 60.62 % of the total observed variance. It seems that a careful choice of items related to the scale might be the reason why we have achieved such satisfactory results. Furthermore, the CFA also showed good fit indices for the current model and the convergent validity of the subscales of the questionnaire was good, with the exception of the self-care subscale. With regard to the latter, all correlations between the items of the self-care subscale and its total score ranged between .21 and .36. Although these results are fair, the values are considerably lower than those of the other subscales. One explanation might be that the items of the self-care subscale all reflect different aspects of self-care (e.g., following an educational program, following a healthy diet, doing physical activities). The internal consistency of the final instrument as assessed by the Cronbach’s alpha coefficient was found to be .80, which reflected an acceptable reliability. In addition, the ICC score indicated an appropriate stability for the questionnaire, as it was examined by 25 women with a 2-week interval (.86). As such, we believe that this newly developed instrument may be especially helpful for healthcare teams to recognize and to plan preventive health strategies that are functional and targeted to specific conditions. The inclusion of seven domains in this instrument further allows health experts to understand how domains in need can be improved.

### Limitations

Although the results of this study demonstrated several benefits, some limitations need to be considered. First, with regard to the sampling, we only interviewed women living in Tehran. As these women are culturally homogeneous, and their viewpoints cannot be generalized to the viewpoint of women living in other cultures. Therefore, it might be interesting for future studies to investigate the reliability and validity of the ASSISTS in a sample of women from different cultural backgrounds and regions. Second, regarding the sampling, the majority of the women in the present study were higher educated (54 %) or employed (66.6 %) women. In future studies, it would be necessary to examine the psychometric properties of the ASSISTS in women from both urban and rural areas with different levels of education and economic status. Third, this study used a minimal criteria sample design to validate the ASSISTS scale. It has to be seen in future studies with a larger sample whether the present results will still hold. Fourth, another limitation of the study is that we used two different samples for our exploratory and confirmatory factor analyses. Although the same procedure was used to collect the data from the women, some background information of the samples was not the same, particularly employment status and education level. This might have impacted the results of our study.

In summary, one of the goals for the century is preventing and controlling chronic diseases such as cancer [48]. To do so, we developed the ASSISTS, which proved to have satisfying psychometric properties. The ASSISTS assesses factors affecting breast cancer preventive behaviors that help to promote women’s health.

## Conclusion

Generally, the study findings suggest that the ASSISTS is a valid and reliable questionnaire to assess factors affecting women’s breast cancer prevention behaviors. Further studies in different populations are recommended to establish stronger psychometric properties for the instrument.

## Appendix

### Attitude

1. My health is OK, which is why I do not think at all that sometime I might develop breast cancer. *(reverse scored)*

2. I feel I will get breast cancer when I do regular breast examinations. *(reverse scored)*

3. If I get breast cancer, my feminine identity will be lost. *(reverse scored)*

18. I don’t feel I can get a clinical breast examination because of its high cost. *(reverse scored)*

19. I don’t have enough time to get preventive care for breast cancer. *(reverse scored)*

21. I don’t like to do breast examinations because I am afraid to find out something is wrong. *(reverse scored)*

22. I am embarrassed removing my clothes in front of others during the breast examination. *(reverse scored)*

23. I don’t feel I need to do breast examinations because I don’t have any problems with my breasts. *(reverse scored)*

### Motivation

4. Maintaining a healthy life is extremely important to me.

5. I am motivated to do breast care because I believe that my life is God’s gift.

6. I am going to carry out breast care activities, which are one of my main health responsibilities.

### Self-efficacy

7. I can keep my healthy behaviors and eating habits even if they are difficult.

8. I am sure that I could find a breast lump by performing BSE correctly.

9. I am able to make decisions about routine mammograms in order to maintain my breast health.

### Supportive systems

10. My family members encourage me to practice the recommended care for improving my breast health.

11. All health professionals help me to increase certain skills to stay healthy.

12. My family members pay attention and give me good advice about breast cancer prevention.

13. I have friends who encourage me to get follow-up health preventive care even if I am not attentive enough.

14. I am covered by insurance to pay the cost of breast checkups.

### Information seeking

15. I am going to get new information and skills to improve my health related to breast cancer.

16. I can get follow-up on new educational programs related to breast cancer from the mass media.

17. Having a suitable relationship with others helps me to share information on breast cancer prevention.

20. I talk to my health care provider about how to perform self-monitoring even if I have difficulty understanding him or her.

### Self-care

24. Because of my body build, I do any care activities that I need with regard to breast cancer.

25. I would participate in a follow-up health care education program that was held in a health center even if I were afraid to talk to my health care provider.

26. To reduce the risk of breast cancer, I try to keep my height and weight proportional.

27. I do moderate physical activity (walking, bicycling, swimming, etc.) at least 30 min each day in order to reduce the risk of breast cancer.

28. I try to have a healthy diet (low-fat, vegetables, fruit…) in order to keep my health and prevent breast cancer.

29. I get a breast checkup at least once a year according to my health care provider’s recommendation (physician, midwife, nurse…).

30. I will talk to my health care provider if I discover a tumor through self-examination by myself.

### Stress-management

31. I try to avoid negative thoughts about breast cancer even if I am afraid that I may have cancer.

32. I use several approaches like relaxation, yoga, reading the Quran, prayer and positive thinking in order to manage daily stress.

33. I have balance in my daily life between rest and work time even if I am tired.
