# Erratum to: development and psychometric testing of a new instrument to measure factors influencing women’s breast cancer prevention behaviors (ASSISTS)

**DOI:** 10.1186/s12905-016-0331-5

**Published:** 2016-08-10

**Authors:** Maryam Khazaee-Pool, Fereshteh Majlessi, Ali Montazeri, Tahereh Pashaei, Ali Gholami, Koen Ponnet

**Affiliations:** 1 Department of Health Education and Promotion, School of Health, Zanjan University of Medical Sciences, Parvin Etesami Street, P.O. Box: 451578-6349, Zanjan, Iran; 2 Department of Health Education and Promotion, School of Health, Tehran University of Medical Sciences, P.O. Box 15875-6951, Tehran, Iran; 3 Mental Health Research Group, Health Metrics Research Center, Institute for Health Sciences Research, ACECR, Tehran, Iran; 4 Social Determinants of Health Research Center, Kurdistan University of Medical Sciences, Sanandaj, Iran; 5 Department of public health, School of Health, Kurdistan University of Medical Sciences, Sanandaj, Iran; 6 Department of Public Health, Neyshabur University of Medical Sciences, Neyshabur, Iran; 7 Department of Epidemiology, School of Public Health, Iran University of Medical Sciences, Tehran, Iran; 8 Department of Communication Studies and Sociology, University of Antwerp, Antwerp, Belgium; 9 Higher Institute for Family Sciences, Odisee, Brussels, Belgium; 10 Antwerp Maritime Academy, Antwerp, Belgium

## Erratum

Following publication of the original article [1] it was brought to our attention that the third author’s family name had been incorrectly spelt. Please note that the third author’s family name is Montazeri and not Montazaeri.

This has now been updated on the BioMed Central website.
